# Virtual and Augmented Reality in Undergraduate Medical Education in Psychiatry: A Systematic Review

**DOI:** 10.1111/tct.70128

**Published:** 2025-06-18

**Authors:** Joanne Rodda, Hanna Mansi, Jacob Fernando‐Sayers, Sharna Bennett, Sukhi Shergill

**Affiliations:** ^1^ Kent and Medway Medical School Canterbury Kent UK; ^2^ Kent and Medway NHS and Social Care Partnership Trust Maidstone Kent UK; ^3^ Maidstone and Tunbridge Wells NHS Trust Maidstone Kent UK

**Keywords:** artificial intelligence, augmented reality, medical student, mental health, psychiatry, simulation, virtual reality

## Abstract

**Background:**

Simulation is widely used in medical education in all specialties; in psychiatry, it usually relies on standardised patients played by actors. Virtual and augmented reality (VR and AR) have the potential to provide standardised and replicable clinical experiences for learners.

**Aims:**

The aim of this study was to evaluate the available literature regarding the use of VR and AR simulation in undergraduate medical education in psychiatry.

**Methods:**

The review was registered on PROSPERO (CRD42024527726) and followed PRISMA guidelines. Three electronic databases were searched using a pre‐designed search string for studies of VR and AR in undergraduate medical student psychiatry education. Primary studies of any design were included. Two authors independently screened all references and extracted data. Learning methods and outcome measures were reported according to Kirkpatrick's training evaluation model. Methodological quality was evaluated using standardised tools.

**Results:**

Searches yielded 7550 references, of which 19 studies from nine different countries were included. Learner satisfaction was generally positive, particularly with higher fidelity simulations. Fewer studies investigated changes in knowledge and skills; some reported improvements, which were often self‐reported by students. Positive changes in learner attitudes, especially empathy and stigma reduction, were also reported. Most studies were based on single interventions.

**Conclusions:**

VR and AR simulation may be a useful addition to undergraduate psychiatry curricular teaching. However, significant gaps remain, including lack of long‐term outcome data, limited evaluation of behavioural change and predominance of single‐exposure interventions. Further research of the broader inclusion of VR and AR into teaching programmes will help to establish their value.

## 1 Introduction

Simulation has become widely accepted as a valuable tool in medical education and is increasingly used in all medical specialties to help bridge the gap between theoretical and clinical application of knowledge, skills and attitudes [1]. In comparison to learning in ‘real world’ clinical settings, simulation has the advantage of providing standardised and controllable exposure to clinical scenarios in which students can learn, make mistakes and try different approaches to the same scenario in repeated trials. In psychiatry, simulation most often involves the use of standardised patients who are usually actors. While simulation aims to provide a ‘safe space’, learners may still experience anxiety or performance pressure, highlighting the importance of ensuring psychological safety.

Systematic reviews and meta‐analyses of simulation methods in psychiatry clinical teaching have reported overall effectiveness, acknowledging the need for caution in interpreting findings due to the heterogeneity of studies [2, 3]. Developments in technology, combined with the rapid increase in remote learning triggered by the COVID‐19 pandemic, have led to a growing interest in digital education in the training of doctors, and in particular virtual reality (VR), including interactions with ‘virtual patients’ (VP, computer simulations of patients) [4]. Virtual reality involves a computer‐generated simulation of a visual or other sensory environment with which the subject can interact. Less immersive forms of virtual reality involve an image on a computer screen, while VR headsets provide an immersive three‐dimensional world. Augmented reality (AR) is a related concept that involves the overlay of virtual elements into a real‐world environment [5].

*Developments in technology, combined with the rapid increase in remote learning triggered by the COVID‐19 pandemic, have led to a growing interest in digital education in the training of doctors, and in particular virtual reality*.

The use of VR and AR in psychiatric education shares many of the advantages and disadvantages of simulation training in general. These teaching modalities can simulate important clinical scenarios that students are not able to access in clinical placements. Virtual patients also allow trainees to pause and consider their questions and/or answers and can enable independent learning of clinical skills. Standardised content also ensures that all students within a given course have access to similar learning opportunities. Financial costs and demands on teaching time are likely to be lower for VR and AR simulations, especially when the ongoing cost of actors is considered. Disadvantages of virtual patients are that at present they are clearly artificially generated, have a limited range of responses, may not recognise questions posed by the learner and do not allow for non‐verbal communication [6]. Just as with psychiatry simulation training in general, some students may find it difficult to ‘suspend disbelief’, and this may be more the case in VR and AR than with the use of actors as real‐life standardised patients.

Despite the growing interest in simulation‐based education, the role of VR and AR in undergraduate psychiatry training remains relatively unexplored. Existing reviews have addressed simulation methods in general or wider learner groups, leaving a gap in understanding their application specifically in the context of undergraduate medical education in psychiatry. Furthermore, rapid technological advancements, including greater accessibility of VR/AR hardware, improvements in the realism of simulations and the growing use of natural language processing, have expanded the potential for immersive learning experiences. Given these developments, a systematic review is timely to synthesise current evidence and identify priorities for future research and implementation. The review focuses on undergraduate medical education because medical students are at an early stage of clinical training, where immersive technologies may provide valuable opportunities for exposure to psychiatric scenarios that are more difficult for them to access in limited clinical placement time.

*Rapid technological advancements, including greater accessibility of VR/AR hardware, improvements in the realism of simulations and the growing use of natural language processing, have expanded the potential for immersive learning experiences*.

This study is a systematic review of the available literature related to the use of VR and AR simulation in undergraduate medical education in psychiatry, including immersive and screen‐based methods. Our primary research question was: What is the current evidence regarding the use of virtual and augmented reality (VR and AR) in undergraduate medical education in psychiatry? Our subsidiary questions were the following: (1) Regarding which psychiatric conditions has the use of VR and AR been investigated in undergraduate medical education in psychiatry? (2) How does the use of VR and AR affect educational outcomes, including *reaction* (student experience of learning), *knowledge* (understanding of key facts and concepts), *skills* (development of practical clinical competencies), attitudes (relevant views and perceptions related to psychiatry), *behaviours* (application of learning in real‐world settings) and *patient impact* (measurable impact of the learning on patient outcomes)?

*This study is a systematic review of the available literature related to the use of VR and AR simulation in undergraduate medical education in psychiatry*.

## 2 Methods

The review was registered on PROSPERO (CRD42024527726). A systematic review was performed and reported according to the Preferred Reporting Items for Systematic Review and Meta‐analysis (PRISMA) 2020 statement [7].

### 2.1 Eligibility Criteria

Studies that evaluated VR and AR simulation in mental health‐related education or training in undergraduate medical students were eligible for inclusion. Inclusion and exclusion criteria are outlined in Table 1.

**TABLE 1:** Inclusion and exclusion criteria for studies.

Inclusion criteria	Exclusion criteria
Studies including undergraduate medical students Studies using VR or AR for educational purposes^a^ Primary studies of any design Published in peer‐reviewed journal Studies of education regarding mental health Relevant ethical approval obtained	Studies in languages other than English Editorials, conference abstracts and opinion pieces Data from presentations, conference proceedings, non‐peer‐reviewed literature, dissertations and theses

Abbreviations: AR = augmented reality; VR = virtual reality.

^a^ Educational purposes were defined according to Kirkpatrick's scale levels [8].

*Studies that evaluated VR and AR simulation in mental health‐related education or training in undergraduate medical students were eligible*.

### 2.2 Search Strategy and Study Selection

Three electronic databases (EMBASE, MEDLINE and PsychInfo) were searched from their inception to 3 September 2024. These databases were selected to ensure comprehensive coverage of biomedical, clinical and health education research, as well as psychological and behavioural sciences research. Search terms were developed following familiarisation with the literature, pilots of different combinations of search terms and discussion with an experienced research librarian.

EMBASE, Medline and PsychInfo were searched via Ovid using multipurpose searches (.mp; includes title, original title, abstract, subject heading, name of substance and registry word fields) and subject headings where these existed. Three blocks of searches were combined using the Boolean operator AND. The first block combined terms for virtual and augmented reality and computer simulation using the Boolean operator ‘OR’. The second block combined a broad range of both general and specific terms for mental illness. The third block combined terms related to education and training. The final search terms are detailed within the Data S1.

*EMBASE, Medline and PsychInfo were searched*.

Backward citation searching was performed by manually reviewing the references of all full reports obtained and those of related reviews and other articles. Forward citation searching of all included reports was performed using Embase and PubMed.

All records were screened against the eligibility criteria by two independent reviewers, and any discrepancies were reviewed by a third party prior to report inclusion.

### 2.3 Data Extraction

A data extraction tool was designed using Microsoft Excel following familiarisation with relevant literature. Two authors (J.R. and H.M. or J.F.) independently extracted data, manually, from each report meeting the eligibility criteria. Any discrepancies were discussed and resolved prior to reporting in the data extraction tool.

Data were collected regarding the year and country of publication, study objective, participant source/descriptor and number, study design, comparator groups, simulation format (VR/AR and details), subject taught, use of adjunctive pedagogical strategies (defined as any additional educational activities or materials used alongside the VR or AR intervention, for example, supplemental reading materials, lectures and videos), outcome measures and study results. The type of learning in each study (student reaction, knowledge, skills, attitudes, behaviours and patient impact) was recorded based on Kirkpatrick's training evaluation model [8], in keeping with World Health Organisation (WHO) methodology for evaluation of training [9]. Our definitions of these learning levels in this context are summarised in Table 2. Kirkpatrick's training evaluation model was selected to provide a structured and well‐established framework for categorising educational outcomes, supporting consistency in data extraction and synthesis. This approach minimised the risk of post hoc bias in outcome selection and offered clarity for data interpretation and reporting.

**TABLE 2:** Definitions used in this review for different learning levels, based on Kirkpatrick's training evaluation model [8].

Student reaction	Learner satisfaction and engagement; how students perceive the usefulness of the learning experience and whether they found it enjoyable, engaging or relevant. Includes perceptions of realism of simulations, perceived changes in confidence and overall satisfaction.
Knowledge	Changes in understanding of essential concepts, facts and principles related to the taught content
Skills	Development or improvement in practical clinical competencies, for example interviewing techniques and diagnostic reasoning
Attitudes	Changes in learners' perspectives, empathy or views towards people living with mental illness, mental illness in general and psychiatry as a specialty
Behaviours	Observable changes that demonstrate application of learned knowledge, skills and attitudes in real‐world environments
Patient impact	Any measurable effect of the educational interventions on actual patient outcomes

The nature, type and potential impact on overall findings of any missing or unclear information were recorded. The methodological quality of the studies included in the review was evaluated using the Cochrane Risk of Bias Tool 2.0 (ROB) [10] for randomised controlled trials and the Medical Education Research Study Quality Instrument (MERSQI) [11] for all studies.

### 2.4 Synthesis

A narrative synthesis was undertaken by J.R. and J.F. to summarise and integrate the findings from the included studies. The data extracted were reviewed, and studies were organised by psychiatric condition, type of VR or AR intervention, with mapping of reported outcomes to the research questions. Quantitative findings, such as statistical significance and effect estimates where available, were summarised descriptively. Qualitative findings, including learner experiences and changes in attitudes, were synthesised thematically to identify common patterns across studies. This approach enabled the integration of diverse study designs and outcome measures, allowing for a coherent summary of the evidence base.

*A narrative synthesis was undertaken*.

## 3 Results

### 3.1 Search Results

**FIGURE 1:**
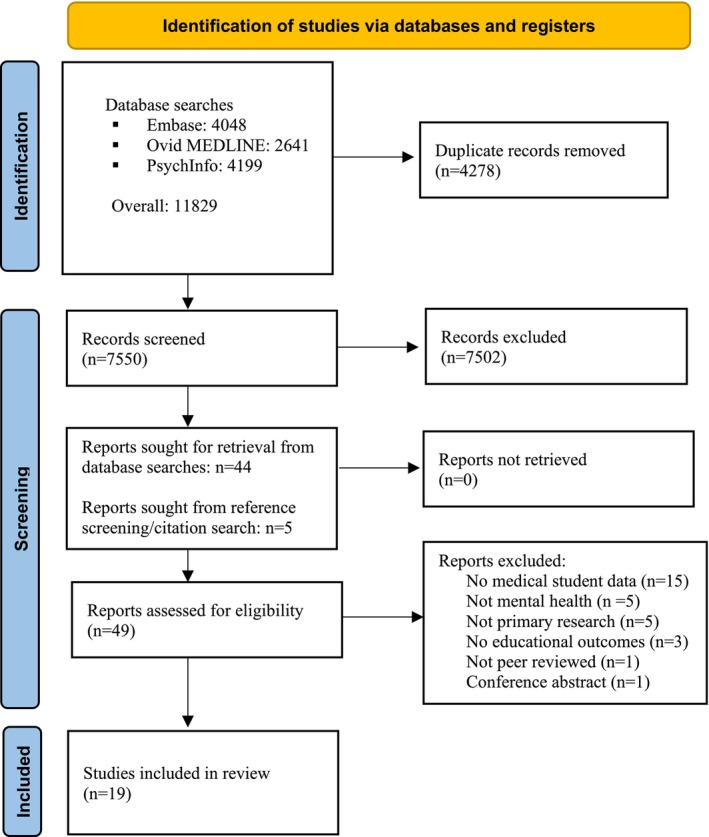
Flow diagram of study selection procedure. AR = augmented reality; VR = virtual reality.

The study selection process is illustrated in Figure 1. The search identified 7550 unduplicated records, all of which were screened using title and abstract. Of these, 44 reports were sought for retrieval. Five further reports were identified through citation searching. Nineteen studies met inclusion criteria. Reasons for study exclusion are provided in Table S1.

*Nineteen studies met inclusion criteria*.

### 3.2 Study Characteristics

Study characteristics are reported in Table S2. Of the 19 studies included, eight were from the United States [6, 12, 13, 14, 15, 16, 17, 18], three were from the United Kingdom [19, 20, 21], two were from Australia [22, 23] and one each was from Brazil [24], France [25], Iran [26], Ireland [27], Japan [28] and Malaysia [29]. Four studies were RCTs [12, 14, 15, 26], all of which were found to be at high risk of bias due to factors including randomisation process, lack of blinding and selection of the reported result. Methodological quality for all studies was assessed using MERSQI scores, which are provided in Table S2 and ranged from 5 to 14.5. Only seven studies had a MERSQI score greater than 12 [12, 13, 14, 15, 18, 25, 27].

*Only seven studies had a MERSQI score greater than 12*.

Almost all studies recruited participants through advertisement or other forms of invitation, with some exceptions where whole cohorts were recruited [6, 18, 26, 28]. Eight studies recruited fewer than 50 participants [16, 17, 18, 20, 21, 24, 25, 29], five included 50–100 [6, 14, 15, 22, 28], three included 100–150 [12, 19, 26] and three included over 150 [13, 23, 27].

Most studies used a single intervention, with one key exception where the intervention took place over a period of 3 months [27].

### 3.3 VR and AR Methods Employed

Several studies used immersive VR headsets to create a simulation of the symptoms or experience of a particular condition, including dementia [19, 22, 23] and schizophrenia [26]. In the only study using AR, Silva et al. attempted to recreate positive symptoms of schizophrenia [24].

Many studies used methods where on‐screen VPs responded to students' questions from a bank of programmed answers and required no facilitator, often providing automated feedback to the learner. This included a system in which questions were chosen from a pre‐specified selection by the student, each of which triggered a different response from VPs as a video clip [12, 25, 27] or static image with text response [29]. Video responses also included a three‐dimensional image of a consulting room on the screen and a representation of an electronic patient record [28]. Five studies used typed natural language input [6, 13, 14, 15, 16] and one used voice recognition [17]; all still relied on a limited bank of possible VP responses.

Two studies used Second Life (an online world in which users create and interact using an avatar) to simulate clinical scenarios [20, 21].

### 3.4 Regarding Which Psychiatric Conditions has the Use of VR and AR Been Investigated in Undergraduate Medical Education in Psychiatry?

Studies were identified that investigated VR or AR in dementia [19, 22, 23, 28, 29]; depression [6, 13, 15, 18, 21, 25, 27]; bipolar disorder [13]; alcohol screening, misuse and brief intervention [12, 16, 18]; suicide risk assessment [14, 21]; PTSD [17, 18]; psychosis [20] and schizophrenia [24, 26]. Although these conditions were the basis for the teaching, the purpose of the interventions invariably related to wider learning, notably interview skills and attitudes, which is summarised below.

### 3.5 What Is the Learner Experience of the Use of VR and AR in Undergraduate Medical Education in Psychiatry?

Student experiences of the use of VR and AR were the most widely reported outcomes. In a study of the use of VR to simulate the experience of dementia in 150 second‐year medical students, almost all students responded that the VR experience affected them emotionally (87%), had made them consider how they would approach patients (94%) and had given them a better understanding of impact of the condition on patients and families (91%) [19]. Another reported that the percentage of students responding that web resources were useful for their learning increased from 20% at baseline to 76% post‐intervention [27].

Several studies investigated the student perception of how accurately the virtual environment or VP mimicked reality, with results indicating that this was an area that interventions fell short. In one study, 55% felt that they were ‘virtually interviewing a patient’ [27], while another reported similarly neutral responses regarding their satisfaction with the interaction with the virtual patient [15]. In one small study, 17/18 students responded that the VP simulation of real life was excellent, but the majority preferred watching videos over a typing‐interface VP as a learning tool [14]. In a study using voice recognition with pre‐recorded VP responses based on an algorithm, some students reported that the VP was frustrating to interview [17]. In contrast, students in a small study using a similar VP form reacted positively regarding the use of VP as an educational tool, ease of use and realism [25]. In another study using an online VP, students described the interaction as unrealistic and the user interface as outdated, unattractive and difficult to navigate [29]. Free‐response feedback regarding the experience of using Second Life was more critical than positive [20].

Studies using more immersive interventions [21, 24, 26] reported higher levels of perceived student engagement with the virtual environment.

*Studies using more immersive interventions [* 21, 24, 26 *] reported higher levels of perceived student engagement with the virtual environment*.

### 3.6 How Does the Use of VR and AR Affect Attitudes, Knowledge, Skills, Behaviours and Patient‐Related Outcomes?

#### 3.6.1 Attitudes

Attitudes were investigated in several studies whose outcomes included changes in empathy and/or stigma. This was studied in the context of dementia [19, 22, 23], depression [13, 25] and in psychosis [24, 26]. All of these studies reported significant improvements in empathy and/or stigma or qualitative data from focus groups that was supportive of the efficacy of the intervention [22].

#### 3.6.2 Knowledge

Three studies assessed knowledge of psychiatric conditions using assessments with SPs. A non‐randomised trial reported increased post‐test knowledge scores in the intervention group (*p* = 0.01) [28]. One study reported improved knowledge on the simulation topics (alcohol misuse, depression and PTSD) and general psychiatry after completion of a computer simulation (*p* = 0.005, *p* = 0.025) [18]. However, a further study reported no change in PTSD knowledge pre‐ and post‐intervention [17].

#### 3.6.3 Skills

Five studies examined clinical skills changes from VR interventions [12, 14, 15, 16, 17]. One study found significant improvement in alcohol screening skills using SPs, with higher scores in the intervention group (*p* < 0.001) after a 3‐month VP intervention [12]. Foster et al. assessed suicide risk skills in 67 students using SPs in an RCT. Small effect sizes favoured the VP intervention, but no significant differences in clinical skills were found [14]. In another study by Foster et al., students using VPs showed significantly greater empathy in SP interviews (*p* = 0.027) [15]. Hayes‐Roth et al. reported 100% improvement in pre‐training skills with the STAR VP, achieving 89% correct responses versus 61% and 50% in E‐Book and control groups [16]. Most studies found significant post‐intervention increases in skills and knowledge, except one, which reported no change in knowledge [17].

#### 3.6.4 Behaviours and Patient‐Related Outcomes

No studies were identified that studied outcomes related to behaviours or patient‐related outcomes.

## 4 Discussion

This systematic review provides a comprehensive analysis of published studies reporting on the use of VR and AR in undergraduate medical education in psychiatry. The findings highlight the potential of these technologies to enhance learning, but also some key limitations. Nineteen studies were identified from nine different countries, including four RCTs.

Most studies consisted of pre‐ and post‐test evaluations of a one‐off VR/AR intervention, with only one study assessing a longer term educational intervention. Only one study of AR was identified, with immersive VR used in four studies. Most of the identified studies used non‐immersive environments, including text‐based or voice‐based interactions with video or still images of VPs. Clinical scenarios used within the studies predominantly focused on dementia, depression or alcohol screening; however, studies using scenarios focusing on suicide risk assessment, bipolar affective disorder, PTSD and schizophrenia/psychosis were also identified.

*Most of the identified studies used non‐immersive environments*.

The methodological quality of the included studies was generally low, with many studies employing small sample sizes, non‐validated outcome measures and predominantly self‐reported data regarding satisfaction with the VP without additional, more impactful outcomes. Furthermore, all included randomised controlled trials were assessed as having a high risk of bias, and most studies had low MERSQI scores, indicating potential weaknesses in study design and reporting. The lack of blinding, incomplete outcome data, and variable fidelity (degree of realism, immersion and replication of the authentic environment) of interventions further limit the reliability and generalisability of the findings. The heterogeneity of study designs and outcome measures also complicated synthesis and comparison across studies.

Students generally reported positive experiences of VR and AR activities. In general, the positivity of students' self‐reported reactions to the simulations correlated with the fidelity of the VPs and VR/AR used within a study. A key limitation of many simulations was the dependence on a finite number of possible responses to questions asked by students; this limits meaningful exploration of interview skills and could make it difficult for many students to suspend disbelief and fully engage in the simulation. In higher fidelity simulations, students reported feeling as though they were virtually interviewing a real patient and gave high Likert ratings for the realism of sound/visual effects and sense of immersion in reality. Negative feedback in higher fidelity simulations predominantly pertained to the user experience, such as platform speed, navigation ease/speed and rigidity of VP responses. In lower fidelity simulations, students preferred videos over typing‐interface VPs, and some students reported frustration during the interaction with the VP in the studies that used voice recognition.

*Students generally reported positive experiences of VR and AR activities*.

Overall, students had positive reactions to the use of VR/AR as a learning tool in psychiatry, as well as positive post‐test outcomes. Little robust evidence is available, however, to compare student reactions and learning outcomes from VP interactions with those from real‐world patients or simulated patients. Furthermore, no studies directly elicited students' opinions on VR/AR simulation as a replacement for real‐world learning opportunities; their positive responses are therefore likely to represent their views on this teaching modality solely as an adjunct to real‐world clinical exposure.

*Overall, students had positive reactions to the use of VR/AR as a learning tool in psychiatry*.

Interest in VR and AR simulation as educational tools is growing. However, there is limited literature available regarding its use in undergraduate education in psychiatry. Nevertheless, the positive reactions and outcomes from VR and AR teaching methods that are highlighted in this review are consistent with broader reviews of simulation training in medical and nursing education in psychiatry [2, 3], and of VR in undergraduate medical education as a whole [4]. The findings are also consistent with the literature on VR and AR as an education tool more broadly. For example, VR has consistently been demonstrated to be an effective means of improving empathy in carers, regardless of the condition being studied [30]. Of important note, interventions focused on empathy may be particularly relevant in undergraduate medical education, because it has consistently been shown to diminish from year 3 of training onward [31, 32].

*There is limited literature available regarding its use in undergraduate education in psychiatry*.

Despite the limited evidence base and the methodological limitations of many studies, VR and AR technologies appear to hold promise as supplementary tools within psychiatry education, particularly for addressing challenges in clinical placement availability and providing safe environments for students to practise managing complex or sensitive scenarios. It is likely that the optimal use of these technologies will be via their careful integration into curricula as adjunctive learning tools. This may include their use in preparatory activities prior to clinical exposure, structured clinical skills‐building exercises, and post‐placement consolidation of clinical learning. Collectively, these approaches can support the meaningful incorporation of VR and AR technologies into psychiatric teaching programmes, broadening experiential learning opportunities and addressing constraints on clinical teaching capacity.

One of the key limitations identified in this review was the restricted interactivity of many VR and AR interventions, which were limited by a finite bank of pre‐programmed responses. This limited the realism of simulations and the opportunity for learners to engage in interactions that they could perceive as dynamic, authentic clinical conversations. The integration of Natural Language Processing (NLP) technologies may help to overcome this obstacle. NLP could enable virtual patients to interpret and respond to free‐text or spoken learner inputs in a more natural and flexible manner, increasing the fidelity of simulations and supporting the development of nuanced communication skills. As such, advances in NLP may play a central role in enhancing the educational value of VR and AR in psychiatric education.

### 4.1 Limitations of the Review

Despite conducting comprehensive searches across multiple databases, there remains the potential for publication bias; for example, studies from grey literature and conferences.

proceedings could have been missed. Additionally, we limited our inclusion to English‐language publications, which may have excluded relevant studies from non‐English speaking contexts. The heterogeneity of study designs, interventions, and outcome measures precluded a quantitative meta‐analysis, meaning findings were synthesised narratively. While we adopted the Kirkpatrick model to provide a structured framework for outcome analysis, this pre‐specified approach may have constrained the identification of emergent themes or novel outcome categories. Finally, while independent review and data extraction processes were used, there remains a risk of selection and interpretation bias.

### 4.2 Conclusion

This systematic review identified and synthesised the available literature regarding the applications of VR and AR in undergraduate psychiatric education, and the educational outcomes associated with these interventions. The findings demonstrate that VR and AR have been employed to teach a range of psychiatric conditions, offering exposure to clinical scenarios that are often difficult to access during medical training. Educational outcomes were generally positive across multiple domains. Learners consistently reported high levels of satisfaction with VR and AR experiences, particularly when simulations offered higher fidelity and interactivity. Several studies demonstrated improvements in knowledge and skills, though these were frequently based on self‐reported measures, and positive shifts in learner attitudes were consistently reported. However, the evidence base remains constrained by methodological limitations, including small sample sizes, high risk of bias, and an absence of studies evaluating behavioural change or patient outcomes. These findings speak to both the current value and the untapped potential of VR and AR in undergraduate psychiatry education. As immersive technologies continue to evolve, they offer promise to enhance medical training by improving access to diverse clinical experiences and supporting the development of essential psychiatric competencies.

## Author Contributions

**Joanne Rodda:** conceptualization, methodology, investigation, data curation, supervision, writing – review and editing, writing – original draft, project administration, formal analysis. **Hanna Mansi:** investigation, data curation, writing – review and editing, formal analysis. **Jacob Fernando‐Sayers:** investigation, writing – review and editing, data curation, formal analysis. **Sharna Bennett:** writing – review and editing, data curation, formal analysis, investigation. **Sukhi Shergill:** conceptualization, methodology, writing – review and editing, supervision, investigation.

## Disclosure

An earlier related review was presented by some of the authors as a poster at the RCPsych Annual Medical Education Conference in October 2023.

The manuscript is an honest, accurate and transparent account of the study being reported, and no important aspects of the study have been omitted.

## Ethics Statement

The authors have nothing to report.

## Conflicts of Interest

The authors declare no conflicts of interest.

## Supporting information


**Appendix S1.** PRISMA 2020 checklist.

**Data S1.** Details of database searches.

**Table S1.** Reasons for exclusion of studies identified from reference screening.

**Table S2.** Summary of study characteristics and results.

## Data Availability

Tables of search strings and outputs are provided in the Supporting Information. Details of the data extracted are in Table S2. An excel spreadsheet with more detailed data regarding evaluation of methodological quality is available on request to the corresponding author.
