# Supplementary material for: Virtual and Augmented Reality in Undergraduate Medical Education in Psychiatry: A Systematic Review
Source: Clin Teach. 2025 Jun 18;22(4):e70128. doi: 10.1111/tct.70128 (PMC12175210; doi:10.1111/tct.70128)
Supplement: Supplementary file 2 — Data S1. Details of database searches. [file TCT-22-e70128-s003.docx]

**Supplementary document 1: Details of database searches**

**PsycInfo** (searched on 03/09/2024)

“Limits applied” = English language only

NOFT = anywhere except full text

| [**Set**](https://www.proquest.com/recentsearches.recentsearchtabview.recentsearchesgridview:toggellistorder?site=psycinfo&t:ac=RecentSearches) | **Search** | **Results** |
| --- | --- | --- |
| **S1** | [noft(virtual reality) OR MAINSUBJECT.EXACT.EXPLODE("Virtual Reality")](https://www.proquest.com/recentsearches.recentsearchtabview.recentsearchesgridview.scrolledrecentsearchlist.checkdbssearchlink:rerunsearch/84E4724A6ADE40DFPQ/None/$N?site=psycinfo&t:ac=RecentSearches)Limits applied | [**18,695**](https://www.proquest.com/recentsearches.recentsearchtabview.recentsearchesgridview.scrolledrecentsearchlist.checkdbssearchlink_0:rerunsearch/84E4724A6ADE40DFPQ/None/$N?site=psycinfo&t:ac=RecentSearches) |
| **S2** | [noft(virtual classrooms) OR MAINSUBJECT.EXACT.EXPLODE("Virtual Classrooms")](https://www.proquest.com/recentsearches.recentsearchtabview.recentsearchesgridview.scrolledrecentsearchlist.checkdbssearchlink:rerunsearch/24C3D9966B924F9EPQ/None/$N?site=psycinfo&t:ac=RecentSearches)Limits applied | [**3,492**](https://www.proquest.com/recentsearches.recentsearchtabview.recentsearchesgridview.scrolledrecentsearchlist.checkdbssearchlink_0:rerunsearch/24C3D9966B924F9EPQ/None/$N?site=psycinfo&t:ac=RecentSearches) |
| **S3** | [noft(virtual environment) OR MAINSUBJECT.EXACT.EXPLODE("Virtual Environment")](https://www.proquest.com/recentsearches.recentsearchtabview.recentsearchesgridview.scrolledrecentsearchlist.checkdbssearchlink:rerunsearch/64D4F7145A2F4113PQ/None/$N?site=psycinfo&t:ac=RecentSearches)Limits applied | [**13,991**](https://www.proquest.com/recentsearches.recentsearchtabview.recentsearchesgridview.scrolledrecentsearchlist.checkdbssearchlink_0:rerunsearch/64D4F7145A2F4113PQ/None/$N?site=psycinfo&t:ac=RecentSearches) |
| **S4** | [noft(augmented reality) OR MAINSUBJECT.EXACT.EXPLODE("Augmented Reality")](https://www.proquest.com/recentsearches.recentsearchtabview.recentsearchesgridview.scrolledrecentsearchlist.checkdbssearchlink:rerunsearch/7BC15D465F404D64PQ/None/$N?site=psycinfo&t:ac=RecentSearches)Limits applied | [**2,203**](https://www.proquest.com/recentsearches.recentsearchtabview.recentsearchesgridview.scrolledrecentsearchlist.checkdbssearchlink_0:rerunsearch/7BC15D465F404D64PQ/None/$N?site=psycinfo&t:ac=RecentSearches) |
| **S5** | [noft(mixed reality)](https://www.proquest.com/recentsearches.recentsearchtabview.recentsearchesgridview.scrolledrecentsearchlist.checkdbssearchlink:rerunsearch/D2AC43C0B855403APQ/None/$N?site=psycinfo&t:ac=RecentSearches)Limits applied | [**2,120**](https://www.proquest.com/recentsearches.recentsearchtabview.recentsearchesgridview.scrolledrecentsearchlist.checkdbssearchlink_0:rerunsearch/D2AC43C0B855403APQ/None/$N?site=psycinfo&t:ac=RecentSearches) |
| **S6** | [noft(computer simulation) OR MAINSUBJECT.EXACT.EXPLODE("Computer Simulation")](https://www.proquest.com/recentsearches.recentsearchtabview.recentsearchesgridview.scrolledrecentsearchlist.checkdbssearchlink:rerunsearch/D53A012EB4AB4931PQ/None/$N?site=psycinfo&t:ac=RecentSearches)Limits applied | [**37,457**](https://www.proquest.com/recentsearches.recentsearchtabview.recentsearchesgridview.scrolledrecentsearchlist.checkdbssearchlink_0:rerunsearch/D53A012EB4AB4931PQ/None/$N?site=psycinfo&t:ac=RecentSearches) |
| **S7** | [noft(computer generated)](https://www.proquest.com/recentsearches.recentsearchtabview.recentsearchesgridview.scrolledrecentsearchlist.checkdbssearchlink:rerunsearch/F77AF1DBFC184EB9PQ/None/$N?site=psycinfo&t:ac=RecentSearches)Limits applied | [**8,756**](https://www.proquest.com/recentsearches.recentsearchtabview.recentsearchesgridview.scrolledrecentsearchlist.checkdbssearchlink_0:rerunsearch/F77AF1DBFC184EB9PQ/None/$N?site=psycinfo&t:ac=RecentSearches) |
| **S8** | [[S1] OR [S2] OR [S3] OR [S4] OR [S5] OR [S6] OR [S7]](https://www.proquest.com/recentsearches.recentsearchtabview.recentsearchesgridview.scrolledrecentsearchlist.checkdbssearchlink:rerunsearch/E634504B02324F51PQ/None/$N?site=psycinfo&t:ac=RecentSearches) | [**57,848**](https://www.proquest.com/recentsearches.recentsearchtabview.recentsearchesgridview.scrolledrecentsearchlist.checkdbssearchlink_0:rerunsearch/E634504B02324F51PQ/None/$N?site=psycinfo&t:ac=RecentSearches) |
| **S9** | [noft(anxiety) OR MAINSUBJECT.EXACT.EXPLODE("Anxiety Disorders")](https://www.proquest.com/recentsearches.recentsearchtabview.recentsearchesgridview.scrolledrecentsearchlist.checkdbssearchlink:rerunsearch/F36A823C78B74F1FPQ/None/$N?site=psycinfo&t:ac=RecentSearches)Limits applied | [**324,118**](https://www.proquest.com/recentsearches.recentsearchtabview.recentsearchesgridview.scrolledrecentsearchlist.checkdbssearchlink_0:rerunsearch/F36A823C78B74F1FPQ/None/$N?site=psycinfo&t:ac=RecentSearches) |
| **S10** | [noft(panic) OR MAINSUBJECT.EXACT.EXPLODE("Panic")](https://www.proquest.com/recentsearches.recentsearchtabview.recentsearchesgridview.scrolledrecentsearchlist.checkdbssearchlink:rerunsearch/EFF699D15471404CPQ/None/$N?site=psycinfo&t:ac=RecentSearches)Limits applied | [**18,746**](https://www.proquest.com/recentsearches.recentsearchtabview.recentsearchesgridview.scrolledrecentsearchlist.checkdbssearchlink_0:rerunsearch/EFF699D15471404CPQ/None/$N?site=psycinfo&t:ac=RecentSearches) |
| **S11** | [noft(PTSD) OR noft(posttraumatic stress disorder) OR MAINSUBJECT.EXACT.EXPLODE("Posttraumatic Stress Disorder")](https://www.proquest.com/recentsearches.recentsearchtabview.recentsearchesgridview.scrolledrecentsearchlist.checkdbssearchlink:rerunsearch/2F7FB080EEED4890PQ/None/$N?site=psycinfo&t:ac=RecentSearches)Limits applied | [**64,956**](https://www.proquest.com/recentsearches.recentsearchtabview.recentsearchesgridview.scrolledrecentsearchlist.checkdbssearchlink_0:rerunsearch/2F7FB080EEED4890PQ/None/$N?site=psycinfo&t:ac=RecentSearches) |
| **S12** | [noft(OCD) OR noft(Obsessive compulsive disorder) OR MAINSUBJECT.EXACT.EXPLODE("Obsessive Compulsive Disorder")](https://www.proquest.com/recentsearches.recentsearchtabview.recentsearchesgridview.scrolledrecentsearchlist.checkdbssearchlink:rerunsearch/DAD0A1E58ABA45A5PQ/None/$N?site=psycinfo&t:ac=RecentSearches)Limits applied | [**28,829**](https://www.proquest.com/recentsearches.recentsearchtabview.recentsearchesgridview.scrolledrecentsearchlist.checkdbssearchlink_0:rerunsearch/DAD0A1E58ABA45A5PQ/None/$N?site=psycinfo&t:ac=RecentSearches) |
| **S13** | [noft(personality disorder) OR MAINSUBJECT.EXACT.EXPLODE("Personality Disorders")](https://www.proquest.com/recentsearches.recentsearchtabview.recentsearchesgridview.scrolledrecentsearchlist.checkdbssearchlink:rerunsearch/B50DE3EB581A4EB9PQ/None/$N?site=psycinfo&t:ac=RecentSearches)Limits applied | [**149,089**](https://www.proquest.com/recentsearches.recentsearchtabview.recentsearchesgridview.scrolledrecentsearchlist.checkdbssearchlink_0:rerunsearch/B50DE3EB581A4EB9PQ/None/$N?site=psycinfo&t:ac=RecentSearches) |
| **S14** | [noft(addiction) OR MAINSUBJECT.EXACT.EXPLODE("Addiction")](https://www.proquest.com/recentsearches.recentsearchtabview.recentsearchesgridview.scrolledrecentsearchlist.checkdbssearchlink:rerunsearch/A0C34EA1F104F95PQ/None/$N?site=psycinfo&t:ac=RecentSearches)Limits applied | [**162,218**](https://www.proquest.com/recentsearches.recentsearchtabview.recentsearchesgridview.scrolledrecentsearchlist.checkdbssearchlink_0:rerunsearch/A0C34EA1F104F95PQ/None/$N?site=psycinfo&t:ac=RecentSearches) |
| **S15** | [noft(alcohol misuse) OR MAINSUBJECT.EXACT.EXPLODE("Alcohol Abuse")](https://www.proquest.com/recentsearches.recentsearchtabview.recentsearchesgridview.scrolledrecentsearchlist.checkdbssearchlink:rerunsearch/384E02C93BF4D92PQ/None/$N?site=psycinfo&t:ac=RecentSearches)Limits applied | [**57,252**](https://www.proquest.com/recentsearches.recentsearchtabview.recentsearchesgridview.scrolledrecentsearchlist.checkdbssearchlink_0:rerunsearch/384E02C93BF4D92PQ/None/$N?site=psycinfo&t:ac=RecentSearches) |
| **S16** | [noft(substance misuse) OR MAINSUBJECT.EXACT.EXPLODE("Substance Abuse and Addiction Measures")](https://www.proquest.com/recentsearches.recentsearchtabview.recentsearchesgridview.scrolledrecentsearchlist.checkdbssearchlink:rerunsearch/9F65143318544AE3PQ/None/$N?site=psycinfo&t:ac=RecentSearches)Limits applied | [**16,464**](https://www.proquest.com/recentsearches.recentsearchtabview.recentsearchesgridview.scrolledrecentsearchlist.checkdbssearchlink_0:rerunsearch/9F65143318544AE3PQ/None/$N?site=psycinfo&t:ac=RecentSearches) |
| **S17** | [noft(eating disorder) OR MAINSUBJECT.EXACT.EXPLODE("Eating Disorders")](https://www.proquest.com/recentsearches.recentsearchtabview.recentsearchesgridview.scrolledrecentsearchlist.checkdbssearchlink:rerunsearch/BD6D1475F2724B94PQ/None/$N?site=psycinfo&t:ac=RecentSearches)Limits applied | [**61,121**](https://www.proquest.com/recentsearches.recentsearchtabview.recentsearchesgridview.scrolledrecentsearchlist.checkdbssearchlink_0:rerunsearch/BD6D1475F2724B94PQ/None/$N?site=psycinfo&t:ac=RecentSearches) |
| **S18** | [noft(Binge Eating Disorder) OR MAINSUBJECT.EXACT.EXPLODE("Binge Eating Disorder")](https://www.proquest.com/recentsearches.recentsearchtabview.recentsearchesgridview.scrolledrecentsearchlist.checkdbssearchlink:rerunsearch/A6CAA51C83684230PQ/None/$N?site=psycinfo&t:ac=RecentSearches)Limits applied | [**8,369**](https://www.proquest.com/recentsearches.recentsearchtabview.recentsearchesgridview.scrolledrecentsearchlist.checkdbssearchlink_0:rerunsearch/A6CAA51C83684230PQ/None/$N?site=psycinfo&t:ac=RecentSearches) |
| **S19** | [noft(Bulimia) OR MAINSUBJECT.EXACT.EXPLODE("Bulimia")](https://www.proquest.com/recentsearches.recentsearchtabview.recentsearchesgridview.scrolledrecentsearchlist.checkdbssearchlink:rerunsearch/971D5FB2BC024AC3PQ/None/$N?site=psycinfo&t:ac=RecentSearches)Limits applied | [**13,346**](https://www.proquest.com/recentsearches.recentsearchtabview.recentsearchesgridview.scrolledrecentsearchlist.checkdbssearchlink_0:rerunsearch/971D5FB2BC024AC3PQ/None/$N?site=psycinfo&t:ac=RecentSearches) |
| **S20** | [noft(ADHD) OR noft(Attention Deficit Hyperactivity Disorder) OR MAINSUBJECT.EXACT.EXPLODE("Attention Deficit Disorder") OR MAINSUBJECT.EXACT.EXPLODE("Attention Deficit Disorder with Hyperactivity")](https://www.proquest.com/recentsearches.recentsearchtabview.recentsearchesgridview.scrolledrecentsearchlist.checkdbssearchlink:rerunsearch/B2654428D09B42DDPQ/None/$N?site=psycinfo&t:ac=RecentSearches)Limits applied | [**46,151**](https://www.proquest.com/recentsearches.recentsearchtabview.recentsearchesgridview.scrolledrecentsearchlist.checkdbssearchlink_0:rerunsearch/B2654428D09B42DDPQ/None/$N?site=psycinfo&t:ac=RecentSearches) |
| **S21** | [noft(autis*) OR MAINSUBJECT.EXACT.EXPLODE("Autism Spectrum Disorders")](https://www.proquest.com/recentsearches.recentsearchtabview.recentsearchesgridview.scrolledrecentsearchlist.checkdbssearchlink:rerunsearch/3756612C8EC14898PQ/None/$N?site=psycinfo&t:ac=RecentSearches)Limits applied | [**97,084**](https://www.proquest.com/recentsearches.recentsearchtabview.recentsearchesgridview.scrolledrecentsearchlist.checkdbssearchlink_0:rerunsearch/3756612C8EC14898PQ/None/$N?site=psycinfo&t:ac=RecentSearches) |
| **S22** | [noft(Dementia) OR MAINSUBJECT.EXACT.EXPLODE("Dementia")](https://www.proquest.com/recentsearches.recentsearchtabview.recentsearchesgridview.scrolledrecentsearchlist.checkdbssearchlink:rerunsearch/34DD0742AC434DBCPQ/None/$N?site=psycinfo&t:ac=RecentSearches)Limits applied | [**121,738**](https://www.proquest.com/recentsearches.recentsearchtabview.recentsearchesgridview.scrolledrecentsearchlist.checkdbssearchlink_0:rerunsearch/34DD0742AC434DBCPQ/None/$N?site=psycinfo&t:ac=RecentSearches) |
| **S23** | [noft(Psychiatry) OR noft(Child Psychiatry) OR noft(Adult Psychiatry) OR noft(Geriatric Psychiatry) OR MAINSUBJECT.EXACT.EXPLODE("Child Psychiatry") OR MAINSUBJECT.EXACT.EXPLODE("Adolescent Psychiatry") OR MAINSUBJECT.EXACT.EXPLODE("Geriatric Psychiatry")](https://www.proquest.com/recentsearches.recentsearchtabview.recentsearchesgridview.scrolledrecentsearchlist.checkdbssearchlink:rerunsearch/FAE3425656084F0APQ/None/$N?site=psycinfo&t:ac=RecentSearches)Limits applied | [**614,951**](https://www.proquest.com/recentsearches.recentsearchtabview.recentsearchesgridview.scrolledrecentsearchlist.checkdbssearchlink_0:rerunsearch/FAE3425656084F0APQ/None/$N?site=psycinfo&t:ac=RecentSearches) |
| **S24** | [[S9] OR [S10] OR [S11] OR [S12] OR [S13] OR [S14] OR [S15] OR [S16] OR [S17] OR [S18] OR [S19] OR [S20] OR [S21] OR [S22] OR [S23]](https://www.proquest.com/recentsearches.recentsearchtabview.recentsearchesgridview.scrolledrecentsearchlist.checkdbssearchlink:rerunsearch/A5244FCB8CDC4A90PQ/None/$N?site=psycinfo&t:ac=RecentSearches) | [**1,255,094**](https://www.proquest.com/recentsearches.recentsearchtabview.recentsearchesgridview.scrolledrecentsearchlist.checkdbssearchlink_0:rerunsearch/A5244FCB8CDC4A90PQ/None/$N?site=psycinfo&t:ac=RecentSearches) |
| **S25** | [noft(Education) OR MAINSUBJECT.EXACT.EXPLODE("Education")](https://www.proquest.com/recentsearches.recentsearchtabview.recentsearchesgridview.scrolledrecentsearchlist.checkdbssearchlink:rerunsearch/4FC503D72B164B37PQ/None/$N?site=psycinfo&t:ac=RecentSearches)Limits applied | [**1,043,697**](https://www.proquest.com/recentsearches.recentsearchtabview.recentsearchesgridview.scrolledrecentsearchlist.checkdbssearchlink_0:rerunsearch/4FC503D72B164B37PQ/None/$N?site=psycinfo&t:ac=RecentSearches) |
| **S26** | [noft(Training) OR MAINSUBJECT.EXACT.EXPLODE("Training")](https://www.proquest.com/recentsearches.recentsearchtabview.recentsearchesgridview.scrolledrecentsearchlist.checkdbssearchlink:rerunsearch/5D0025C0067941FCPQ/None/$N?site=psycinfo&t:ac=RecentSearches)Limits applied | [**455,679**](https://www.proquest.com/recentsearches.recentsearchtabview.recentsearchesgridview.scrolledrecentsearchlist.checkdbssearchlink_0:rerunsearch/5D0025C0067941FCPQ/None/$N?site=psycinfo&t:ac=RecentSearches) |
| **S27** | [noft(Medical education) OR MAINSUBJECT.EXACT.EXPLODE("Medical Education")](https://www.proquest.com/recentsearches.recentsearchtabview.recentsearchesgridview.scrolledrecentsearchlist.checkdbssearchlink:rerunsearch/5068F7181B114CCEPQ/None/$N?site=psycinfo&t:ac=RecentSearches)Limits applied | [**125,026**](https://www.proquest.com/recentsearches.recentsearchtabview.recentsearchesgridview.scrolledrecentsearchlist.checkdbssearchlink_0:rerunsearch/5068F7181B114CCEPQ/None/$N?site=psycinfo&t:ac=RecentSearches) |
| **S28** | [noft(Learning) OR MAINSUBJECT.EXACT.EXPLODE("Learning")](https://www.proquest.com/recentsearches.recentsearchtabview.recentsearchesgridview.scrolledrecentsearchlist.checkdbssearchlink:rerunsearch/F1A9DA1130E4893PQ/None/$N?site=psycinfo&t:ac=RecentSearches)Limits applied | [**1,216,640**](https://www.proquest.com/recentsearches.recentsearchtabview.recentsearchesgridview.scrolledrecentsearchlist.checkdbssearchlink_0:rerunsearch/F1A9DA1130E4893PQ/None/$N?site=psycinfo&t:ac=RecentSearches) |
| **S29** | [noft(Empathy) OR MAINSUBJECT.EXACT.EXPLODE("Empathy")](https://www.proquest.com/recentsearches.recentsearchtabview.recentsearchesgridview.scrolledrecentsearchlist.checkdbssearchlink:rerunsearch/155FB97D12B14480PQ/None/$N?site=psycinfo&t:ac=RecentSearches)Limits applied | [**36,945**](https://www.proquest.com/recentsearches.recentsearchtabview.recentsearchesgridview.scrolledrecentsearchlist.checkdbssearchlink_0:rerunsearch/155FB97D12B14480PQ/None/$N?site=psycinfo&t:ac=RecentSearches) |
| **S30** | [noft(Stigma) OR MAINSUBJECT.EXACT.EXPLODE("Stigma")](https://www.proquest.com/recentsearches.recentsearchtabview.recentsearchesgridview.scrolledrecentsearchlist.checkdbssearchlink:rerunsearch/3576995291C54A35PQ/None/$N?site=psycinfo&t:ac=RecentSearches)Limits applied | [**40,122**](https://www.proquest.com/recentsearches.recentsearchtabview.recentsearchesgridview.scrolledrecentsearchlist.checkdbssearchlink_0:rerunsearch/3576995291C54A35PQ/None/$N?site=psycinfo&t:ac=RecentSearches) |
| **S31** | [noft(Communication) OR MAINSUBJECT.EXACT.EXPLODE("Communication Skills")](https://www.proquest.com/recentsearches.recentsearchtabview.recentsearchesgridview.scrolledrecentsearchlist.checkdbssearchlink:rerunsearch/DCDBF35063A14C80PQ/None/$N?site=psycinfo&t:ac=RecentSearches)Limits applied | [**457,678**](https://www.proquest.com/recentsearches.recentsearchtabview.recentsearchesgridview.scrolledrecentsearchlist.checkdbssearchlink_0:rerunsearch/DCDBF35063A14C80PQ/None/$N?site=psycinfo&t:ac=RecentSearches) |
| **S32** | [noft(Attitudes) OR MAINSUBJECT.EXACT.EXPLODE("Attitudes")](https://www.proquest.com/recentsearches.recentsearchtabview.recentsearchesgridview.scrolledrecentsearchlist.checkdbssearchlink:rerunsearch/C93E00AC6F044D3CPQ/None/$N?site=psycinfo&t:ac=RecentSearches)Limits applied | [**811,957**](https://www.proquest.com/recentsearches.recentsearchtabview.recentsearchesgridview.scrolledrecentsearchlist.checkdbssearchlink_0:rerunsearch/C93E00AC6F044D3CPQ/None/$N?site=psycinfo&t:ac=RecentSearches) |
| **S33** | [noft(Knowledge) OR MAINSUBJECT.EXACT.EXPLODE("Knowledge (General)")](https://www.proquest.com/recentsearches.recentsearchtabview.recentsearchesgridview.scrolledrecentsearchlist.checkdbssearchlink:rerunsearch/C6EEBFD1487948F8PQ/None/$N?site=psycinfo&t:ac=RecentSearches)Limits applied | [**454,987**](https://www.proquest.com/recentsearches.recentsearchtabview.recentsearchesgridview.scrolledrecentsearchlist.checkdbssearchlink_0:rerunsearch/C6EEBFD1487948F8PQ/None/$N?site=psycinfo&t:ac=RecentSearches) |
| **S34** | [noft(Skills)](https://www.proquest.com/recentsearches.recentsearchtabview.recentsearchesgridview.scrolledrecentsearchlist.checkdbssearchlink:rerunsearch/1DF73B0D8E164B2DPQ/None/$N?site=psycinfo&t:ac=RecentSearches)Limits applied | [**318,730**](https://www.proquest.com/recentsearches.recentsearchtabview.recentsearchesgridview.scrolledrecentsearchlist.checkdbssearchlink_0:rerunsearch/1DF73B0D8E164B2DPQ/None/$N?site=psycinfo&t:ac=RecentSearches) |
| **S35** | [noft(Student)](https://www.proquest.com/recentsearches.recentsearchtabview.recentsearchesgridview.scrolledrecentsearchlist.checkdbssearchlink:rerunsearch/9C94B3BC102E4930PQ/None/$N?site=psycinfo&t:ac=RecentSearches)Limits applied | [**741,839**](https://www.proquest.com/recentsearches.recentsearchtabview.recentsearchesgridview.scrolledrecentsearchlist.checkdbssearchlink_0:rerunsearch/9C94B3BC102E4930PQ/None/$N?site=psycinfo&t:ac=RecentSearches) |
| **S36** | [noft(Medical Student)](https://www.proquest.com/recentsearches.recentsearchtabview.recentsearchesgridview.scrolledrecentsearchlist.checkdbssearchlink:rerunsearch/BA82033EE89A4582PQ/None/$N?site=psycinfo&t:ac=RecentSearches)Limits applied | [**54,082**](https://www.proquest.com/recentsearches.recentsearchtabview.recentsearchesgridview.scrolledrecentsearchlist.checkdbssearchlink_0:rerunsearch/BA82033EE89A4582PQ/None/$N?site=psycinfo&t:ac=RecentSearches) |
| **S37** | [[S25] OR [S26] OR [S27] OR [S28] OR [S29] OR [S30] OR [S31] OR [S32] OR [S33] OR [S34] OR [S35] OR [S36]](https://www.proquest.com/recentsearches.recentsearchtabview.recentsearchesgridview.scrolledrecentsearchlist.checkdbssearchlink:rerunsearch/55D97221A5C443FEPQ/None/$N?site=psycinfo&t:ac=RecentSearches) | [**3,019,627**](https://www.proquest.com/recentsearches.recentsearchtabview.recentsearchesgridview.scrolledrecentsearchlist.checkdbssearchlink_0:rerunsearch/55D97221A5C443FEPQ/None/$N?site=psycinfo&t:ac=RecentSearches) |
| **S38** | [[S8] AND [S24] AND [S37]](https://www.proquest.com/recentsearches.recentsearchtabview.recentsearchesgridview.scrolledrecentsearchlist.checkdbssearchlink:rerunsearch/38435599217D4A45PQ/None/$N?site=psycinfo&t:ac=RecentSearches) | [**4,199**](https://www.proquest.com/recentsearches.recentsearchtabview.recentsearchesgridview.scrolledrecentsearchlist.checkdbssearchlink_0:rerunsearch/38435599217D4A45PQ/None/$N?site=psycinfo&t:ac=RecentSearches) |

**Ovid MEDLINE(R) ALL**1946 to September 03, 2024 (searched on 04/09/2024)

[mp = multi-purpose fields searched: title, book title, abstract, original title, name of substance word, subject heading word, floating sub-heading word, keyword heading word, organism supplementary concept word, protocol supplementary concept word, rare disease supplementary concept word, unique identifier, synonyms, population supplementary concept word, anatomy supplementary concept word]

| **#** | **Searches** | **Results** |
| --- | --- | --- |
| 1 | Virtual reality.mp. or Virtual Reality/ | 21791 |
| 2 | Virtual patient.mp. | 1166 |
| 3 | Virtual environment.mp. | 3551 |
| 4 | Augmented reality.mp. or Augmented Reality/ | 5735 |
| 5 | Mixed reality.mp. | 1260 |
| 6 | Computer simulation.mp. or Computer Simulation/ | 224701 |
| 7 | Computer generated.mp. | 9123 |
| 8 | 1 or 2 or 3 or 4 or 5 or 6 or 7 | 258855 |
| 9 | Psychiatric.mp. | 335199 |
| 10 | Mental health.mp. or Mental Health/ | 300115 |
| 11 | Mental illness.mp. or Mental Disorders/ | 203170 |
| 12 | Depression/ or Depression.mp. | 528143 |
| 13 | Mood disorder.mp. or Mood Disorders/ | 21680 |
| 14 | Mania.mp. or Mania/ | 12618 |
| 15 | Bipolar disorder.mp. or Bipolar Disorder/ | 59397 |
| 16 | Schizophrenia/ or Schizophrenia.mp. | 166844 |
| 17 | Psychosis.mp. | 49331 |
| 18 | Psychotic Disorders/ or Psychotic.mp. | 80205 |
| 19 | Suicide.mp. or Suicide/ | 105105 |
| 20 | Anxiety/ or Anxiety.mp. | 338188 |
| 21 | Panic.mp. or Panic/ | 18891 |
| 22 | Agoraphobia.mp. or Agoraphobia/ | 4338 |
| 23 | Stress Disorders, Post-Traumatic/ or Post?traumatic stress disorder.mp. | 51050 |
| 24 | PTSD.mp. | 36720 |
| 25 | Obsessive compulsive disorder.mp. or Obsessive-Compulsive Disorder/ | 22932 |
| 26 | OCD.mp. | 12790 |
| 27 | Personality disorder.mp. or Personality Disorders/ | 48580 |
| 28 | Addiction.mp. | 64123 |
| 29 | Alcohol.mp. | 371387 |
| 30 | Substance misuse.mp. | 3918 |
| 31 | Eating disorder.mp. or "Feeding and Eating Disorders"/ | 28722 |
| 32 | Anorexia nervosa.mp. or Anorexia Nervosa/ | 19878 |
| 33 | Bulimia Nervosa.mp. or Bulimia Nervosa/ | 7515 |
| 34 | Binge eating disorder.mp. or Binge-Eating Disorder/ | 4650 |
| 35 | Attention deficit hyperactivity disorder.mp. or Attention Deficit Disorder with Hyperactivity/ | 47444 |
| 36 | ADHD.mp. | 34672 |
| 37 | Attention deficit disorder.mp. | 37334 |
| 38 | Autism.mp. or Autistic Disorder/ | 74391 |
| 39 | Dementia/ or Dementia.mp. | 172211 |
| 40 | Psychiatry.mp. or Psychiatry/ | 110999 |
| 41 | Child Psychiatry/ | 5872 |
| 42 | Adolescent Psychiatry/ | 3155 |
| 43 | Geriatric Psychiatry/ | 2549 |
| 44 | Geropsychiatry.mp. | 108 |
| 45 | 9 or 10 or 11 or 12 or 13 or 14 or 15 or 16 or 17 or 18 or 19 or 20 or 21 or 22 or 23 or 24 or 25 or 26 or 27 or 28 or 29 or 30 or 31 or 32 or 33 or 34 or 35 or 36 or 37 or 38 or 39 or 40 or 41 or 42 or 43 or 44 | 2117483 |
| 46 | Education.mp. or Education, Medical/ or Education, Medical, Graduate/ or Education, Medical, Undergraduate/ or Education/ | 1122762 |
| 47 | Training.mp. | 648071 |
| 48 | Teaching/ or Teaching.mp. | 229868 |
| 49 | Medical education.mp. | 66446 |
| 50 | Learning/ or Learning.mp. | 652319 |
| 51 | Empathy.mp. or Empathy/ | 35267 |
| 52 | Stigma.mp. | 44054 |
| 53 | Communication.mp. or Communication/ | 477582 |
| 54 | Communication skills.mp. | 15557 |
| 55 | Attitude/ or Attitude?.mp. | 502912 |
| 56 | Knowledge/ or Health Knowledge, Attitudes, Practice/ or Knowledge.mp. | 1072097 |
| 57 | Skills.mp. | 241207 |
| 58 | Student.mp. or Students/ | 191753 |
| 59 | 46 or 47 or 48 or 49 or 50 or 51 or 52 or 53 or 54 or 55 or 56 or 57 or 58 | 3686198 |
| 60 | 8 and 45 and 59 | 2694 |
| 61 | limit 60 to english language | 2641 |

**Embase**1974 to 2024 September 03 (searched on 04/09/2024)

[mp = multi-purpose fields searched: title, book title, abstract, original title, name of substance word, subject heading word, floating sub-heading word, keyword heading word, organism supplementary concept word, protocol supplementary concept word, rare disease supplementary concept word, unique identifier, synonyms, population supplementary concept word, anatomy supplementary concept word]

| **#** | **Searches** | **Results** |
| --- | --- | --- |
| 1 | Virtual reality.mp. or Virtual Reality/ | 37539 |
| 2 | Virtual patient.mp. | 1747 |
| 3 | Virtual environment.mp. | 4376 |
| 4 | Augmented reality.mp. or Augmented Reality/ | 6822 |
| 5 | Mixed reality.mp. | 1378 |
| 6 | Computer simulation.mp. or Computer Simulation/ | 150013 |
| 7 | Computer generated.mp. | 10714 |
| 8 | 1 or 2 or 3 or 4 or 5 or 6 or 7 | 203204 |
| 9 | Psychiatric.mp. | 354929 |
| 10 | Mental health.mp. or Mental Health/ | 445875 |
| 11 | Mental illness.mp. or Mental Disorders/ | 171186 |
| 12 | Depression/ or Depression.mp. | 916220 |
| 13 | Mood disorder.mp. or Mood Disorders/ | 62331 |
| 14 | Mania.mp. or Mania/ | 31742 |
| 15 | Bipolar disorder.mp. or Bipolar Disorder/ | 85968 |
| 16 | Schizophrenia/ or Schizophrenia.mp. | 245025 |
| 17 | Psychosis.mp. | 158271 |
| 18 | Psychotic Disorders/ or Psychotic.mp. | 102728 |
| 19 | Suicide.mp. or Suicide/ | 138718 |
| 20 | Anxiety/ or Anxiety.mp. | 544486 |
| 21 | Panic.mp. or Panic/ | 34013 |
| 22 | Agoraphobia.mp. or Agoraphobia/ | 8007 |
| 23 | Stress Disorders, Post-Traumatic/ or Post?traumatic stress disorder.mp. | 90989 |
| 24 | PTSD.mp. | 48248 |
| 25 | Obsessive compulsive disorder.mp. or Obsessive-Compulsive Disorder/ | 38287 |
| 26 | OCD.mp. | 18988 |
| 27 | Personality disorder.mp. or Personality Disorders/ | 58010 |
| 28 | Addiction.mp. | 162054 |
| 29 | Alcohol.mp. | 725482 |
| 30 | Substance misuse.mp. | 5468 |
| 31 | Eating disorder.mp. or "Feeding and Eating Disorders"/ | 45424 |
| 32 | Anorexia nervosa.mp. or Anorexia Nervosa/ | 26967 |
| 33 | Bulimia Nervosa.mp. or Bulimia Nervosa/ | 15417 |
| 34 | Binge eating disorder.mp. or Binge-Eating Disorder/ | 10604 |
| 35 | Attention deficit hyperactivity disorder.mp. or Attention Deficit Disorder with Hyperactivity/ | 52011 |
| 36 | ADHD.mp. | 50590 |
| 37 | Attention deficit disorder.mp. | 69363 |
| 38 | Autism.mp. or Autistic Disorder/ | 111129 |
| 39 | Dementia/ or Dementia.mp. | 271334 |
| 40 | Psychiatry.mp. or Psychiatry/ | 178036 |
| 41 | Child Psychiatry/ | 23555 |
| 42 | Adolescent Psychiatry/ | 23555 |
| 43 | Geriatric Psychiatry/ | 8052 |
| 44 | Geropsychiatry.mp. | 164 |
| 45 | 9 or 10 or 11 or 12 or 13 or 14 or 15 or 16 or 17 or 18 or 19 or 20 or 21 or 22 or 23 or 24 or 25 or 26 or 27 or 28 or 29 or 30 or 31 or 32 or 33 or 34 or 35 or 36 or 37 or 38 or 39 or 40 or 41 or 42 or 43 or 44 | 3164809 |
| 46 | Education.mp. or Education, Medical/ or Education, Medical, Graduate/ or Education, Medical, Undergraduate/ or Education/ | 1484099 |
| 47 | Training.mp. | 919247 |
| 48 | Teaching/ or Teaching.mp. | 303006 |
| 49 | Medical education.mp. | 286683 |
| 50 | Learning/ or Learning.mp. | 829690 |
| 51 | Empathy.mp. or Empathy/ | 41233 |
| 52 | Stigma.mp. | 62115 |
| 53 | Communication.mp. or Communication/ | 693425 |
| 54 | Communication skills.mp. | 20800 |
| 55 | Attitude/ or Attitude?.mp. | 634438 |
| 56 | Knowledge/ or Health Knowledge, Attitudes, Practice/ or Knowledge.mp. | 1350508 |
| 57 | Skills.mp. | 284276 |
| 58 | Student.mp. or Students/ | 496877 |
| 59 | 46 or 47 or 48 or 49 or 50 or 51 or 52 or 53 or 54 or 55 or 56 or 57 or 58 | 4948817 |
| 60 | 8 and 45 and 59 | 4165 |
| 61 | limit 60 to english language | 4084 |
