# Supplementary material for: Virtual and Augmented Reality in Undergraduate Medical Education in Psychiatry: A Systematic Review
Source: Clin Teach. 2025 Jun 18;22(4):e70128. doi: 10.1111/tct.70128 (PMC12175210; doi:10.1111/tct.70128)
Supplement: Supplementary file 3 — Table S1. Reasons for exclusion of studies identified from reference screening. [file TCT-22-e70128-s004.docx]

**Suplementary table 1: Reasons for exclusion of studies identified from reference screening**

| **Paper** | **Included?** | **Reason for exclusion** |
| --- | --- | --- |
| Albright G, Bryan C, Adam C, McMillan J, Shockley K. Using virtual patient simulations to prepare primary health care professionals to conduct substance use and mental health screening and brief intervention. 2018; Vol. 24, Journal of the American Psychiatric Nurses Association. p. 247–59. | No | Primary health clinicians (not undergraduate medical students) |
| Bahadur AG, Hargreaves F, Antinucci R, Sockalingam S, Abdool PS. Virtual reality simulation for suicide risk assessment training: Prevalence of adverse effects. 2024; Vol. 48, Academic Psychiatry. p. 57–60. | No | Not undergraduate medical student participants |
| Balas JS, Phelps EB, Shaw C, Washington E, Glover CM, Ludwig GA, et al. Leveraging virtual reality to train certified nursing assistants as essential dementia-care personnel in the age of COVID-19. Alzheimer’s & dementia: the journal of the Alzheimer’s Association. 2021;17(Supplement 11) (pp e051128) | No | Not undergraduate medical students participants (nursing) |
| Bard JT, Chung HK, Shaia JK, Wellman LL, Elzie CA. Increased medical student understanding of dementia through virtual embodiment. Gerontology & geriatrics education. 2023;44(2):211–22. | Yes |  |
| Campbell D, Lugger S, Sigler GS, Turkelson C. Increasing awareness, sensitivity, and empathy for Alzheimer’s dementia patients using simulation. Nurse education today. 2021;98:104764. | No | Not undergraduate medical student participants (nursing) |
| Coggins A, Marchant D, Bartels J, Cliff B, Warburton S, Murphy M, et al. Simulation-based medical education can be used to improve the mental health competency of emergency physicians. Australasian Psychiatry. 2020;28(3):354–8. | No | Study of qualified professionals (Not undergraduate medical students) |
| Coleman D, Black N, Ng J, Blumenthal E. Kognito’s Avatar-Based Suicide Prevention Training for College Students: Results of a Randomized Controlled Trial and a Naturalistic Evaluation. Suicide & life-threatening behavior. 2019;49(6):1735–45. | No | Not undergraduate medical student participants |
| Deladisma AM, Cohen M, Stevens A, Wagner P, Lok B, Bernard T, et al. Do medical students respond empathetically to a virtual patient?. American Journal of Surgery. 2007;193(6):756–60. | No | Not mental health specific (communication skills in surgeons) |
| Dupuy L, Micoulaud-Franchi JA, Cassoudesalle H, Ballot O, Dehail P, Aouizerate B, et al. Evaluation of a virtual agent to train medical students conducting psychiatric interviews for diagnosing major depressive disorders. Journal of Affective Disorders. 2020; 263:1-8. | Yes |  |
| Fitzmaurice B, Armstrong K, Carroll V, Dagger D, Gill M. Virtual interviews for students interacting online for psychiatry (VISIOn): A novel resource for learning clinical interview skills. Psychiatric Bulletin. 2007;31(6):218–20. | Yes |  |
| Fleming M, Olsen D, Stathes H, Boteler L, Grossberg P, Pfeifer J, et al. Virtual reality skills training for health care professionals in alcohol screening and brief intervention. Journal of the American Board of Family Medicine. 2009;22(4):387–98. | Yes |  |
| Foster A, Chaudhary N, Kim T, Waller JL, Wong J, Borish M, et al. Using Virtual Patients to Teach Empathy: A Randomized Controlled Study to Enhance Medical Students’ Empathic Communication. Simulation in healthcare : journal of the Society for Simulation in Healthcare. 2016;11(3):181–9. | Yes |  |
| Foster A, Harms J, Ange B, Rossen B, Lok B, Lind D S, et al. Empathetic communication in medical students’ interactions with mental health virtual patient scenarios: a descriptive study using the Empathetic Communication Coding System. Austin Journal of Psychiatry and Behavioural Sciences. 2014; 1(3):6. | Yes  Note: from reference screening/citation searches |  |
| Foster A, Chaudhary N, Murphy J, Lok B, Waller J, Buckley PF. The use of simulation to teach suicide risk assessment to health profession trainees—rationale, methodology, and a proof of concept demonstration with a virtual patient. Academic psychiatry. 2015; 39:620-9. | Yes |  |
| Frey T, Gallimore C. Evaluation of virtual patient cases in a psychiatry elective seminar course. Journal of Pharmacy PracticeConference: 14th Annual Meeting of the College of Psychiatric and Neurologic Pharmacists, CPNP 2011Phoenix, AZ United StatesConference Publication: (var.pagings). 2011;24(2):272–3. | No | Not undergraduate medical student participants (pharmacy) |
| Garcia L, Robitaille A, Bouchard S, Rivard MC, McCleary L. The usability of virtual reality to train individuals in responding to behaviors related to dementia. Frontiers in Dementia. 2024 Jan 8;2:1237127. | No | No specific data regarding medical students |
| Gilmartin-Thomas JF, McNeil J, Powell A, Malone DT, Wolfe R, Larson IC, et al. Impact of a virtual dementia experience on medical and pharmacy students’ knowledge and attitudes toward people with dementia: A controlled study. Journal of Alzheimer's Disease. 2018; 62(2):867-76. | Yes |  |
| Gilmartin-Thomas JF, McNeil J, Powell A, Malone DT, Larson IC, O’Reilly CL, et al. Qualitative evaluation of how a virtual dementia experience impacts medical and pharmacy students’ self-reported knowledge and attitudes towards people with dementia. Dementia. 2020; 19(2):205-20. | yes |  |
| Gilmartin Thomas JFM, Duncan G. Educating students about dementia with virtual-learning experiences. American Journal of Health-System Pharmacy. 2017;74(13):956–7. | No | Not primary research |
| Gormley G, McLaughlin N, Rodgers J, D’arcy J. ‘I’m Sorry Doctor but i Didn’t Hear That.’: Developing a Virtual Reality (Vr) Hearing Impairment Learning Experience for Medical Students. In BMJ Simulation and Technology Enhanced Learning. Conference: 10th Annual Conference of the Association of Simulated Practice in Healthcare. Belfast United Kingdom. 5(Supplement 2) (pp A45); BMJ Publishing Group; 2019. | No  Note: from reference screening/citation searches | Not specific to mental health |
| Gutierrez Maldonado J, FerrerGarcia M, PlaSanjuanelo J, AndresPueyo A, TalarnCaparros A. Virtual reality to train diagnostic skills in eating disorders. Comparison of two low cost systems. Journal of Cyber Therapy and RehabilitationConference: 20th Annual CyberPsychology, CyberTherapy and Social Networking Conference, CYPSY 2015San Diego, CA United States. 2015;8(1):25. | No | Not undergraduate medical student participants |
| Gutierrez-Maldonado J, Ferrer-Garcia M, Pla J, Andres-Pueyo A. Virtual humans and formative assessment to train diagnostic skills in bulimia nervosa. Studies in Health Technology & Informatics. 2014;199:30–4. | No | Not undergraduate medical student participants |
| Hayes-Roth B, Amano K, Saker R, Sephton T. Training brief intervention with a virtual coach and virtual patients. 2004; Vol. 2, Annual Review of CyberTherapy and Telemedicine. p. 85–95. | Yes |  |
| Jachna JS, Powsner SM, McIntyre PJ, Byck R. Teaching consultation psychiatry through computerized case simulation. Academic Psychiatry. 1993 Mar;17(1):36-42. | Yes |  |
| Jensen ES, Omdahl M. Usability of virtual reality in inpatient psychiatry: Evaluation of an attempt to reduce coercion. In European Psychiatry. Conference: 28th European Congress of Psychiatry, EPA 2020. Virtual. 63(Supplement 1) (pp S132-S133); Cambridge University Press; 2020. | No | Not undergraduate medical student participants |
| Jensen RAA, Musaeus P, Pedersen K. Virtual patients in undergraduate psychiatry education: a systematic review and synthesis. Advances in health sciences education : theory and practice. 2024;29(1):329–47. | No | Not primary research |
| Jones C, Khalil D, Mander K, Yeoh A, Moro C. Providing dementia education with augmented reality: a health sciences and medicine feasibility pilot study. Research in Learning Technology. 2022 May 2;30:2668. | No | Not clinical mental health – focused on anatomy (Not specific to mental health) |
| Kenny PG, Parsons TD, Rizzo A. A comparative analysis between experts and novices interacting with a virtual patient with PTSD. Annual Review of CyberTherapy and Telemedicine. 2009;7(1):122–4. | No | Paper about technical aspects  (no educational outcomes) |
| Lin CC, Wu WC, Liaw HT, Liu CC. Effectiveness of a virtual patient program in a psychiatry clerkship. Medical education. 2012 Nov;46(11):1111–2. | No | Not peer reviewed journal article |
| Marques AJ, Veloso PG, Araujo M, Almeida RS de, Correia A, Pereira J, et al. Impact of a Virtual Reality-Based Simulation on Empathy and Attitudes Toward Schizophrenia. Frontiers in Psychology. 2022;13:814984. | No | Not undergraduate medical student participants |
| Matsumura Y, Shinno H, Mori T, Nakamura Y. Simulating clinical psychiatry for medical students: a comprehensive clinic simulator with virtual patients and an electronic medical record system. Academic Psychiatry. 2018; 42:613-21. | Yes |  |
| Mousavi B, ZareBidaki M, Ehteshampour A. PAPER: Virtual reality-based training in psychiatry: Prospective and challenges in low- and middle-income countries. In Early Intervention in Psychiatry. Conference: 14th International Conference on Early Intervention in Mental Health, IEPA 2023. Lausanne Switzerland. 17(Supplement 1) (pp 110-111); John Wiley and Sons Inc; 2023. | No | Conference abstract |
| Mudunkotuwe J, Mannali V, Henry J, Clift J, Strickland P. Digitised remote delivery of simulation in psychiatry during the pandemic and for the future. In European Psychiatry. Conference: 30th th European Congress of Psychiatry, EPA 2022. Virtual. 65(Supplement 1) (pp S199); Cambridge University Press; 2022. | No | Not primary research |
| O’Brien KH, Fuxman S, Humm L, Tirone N, Pires WJ, Cole A, Grumet JG. Suicide risk assessment training using an online virtual patient simulation. Mhealth. 2019;5. | No | Not undergraduate medical student participants |
| Ouanes S, Larnaout A, Jouini L. Use of modern technology in psychiatry training in a middle-income country. Asia-Pacific Psychiatry. 2021;13(4) (pagination):Article Number: e12496. Date of Publication: December 2021. | No  Note: from reference screening/citation searches | Not primary research |
| Pantziaras I, Fors U, Ekblad S. Innovative training with virtual patients in transcultural psychiatry: The impact on resident psychiatrists’ confidence. PLoS ONE. 2015;10(3) (pagination):Article Number: e0119754. Date of Publication: 20 Mar 2015. | No | Not undergraduate medical student participants |
| Parsons K, Parsons T, Pataki C, Paton M, St George C, Sugar J, Rizzo A. Virtual Justina: A PTSD virtual patient for clinical classroom training. Annu. Rev. CyberTherapy Telemedicine. 2008;6. | No  Note: from reference screening/citation searches | Paper about the module used (no educational outcomes) |
| Pataki C, Pato MT, Sugar J, George CS, Kenny P. Virtual patients as novel teaching tools in psychiatry. Academic Psychiatry. 2012; 36(5):398. | Yes |  |
| Rampling J, O'Brien A, Hindhaugh K, Woodham L, Kavia S. Use of an online virtual environment in psychiatric problem-based learning. The Psychiatrist. 2012; 36(10):391-6. | Yes |  |
| Shah H, Londino D, Lind SD, Foster A. Interactive virtual-patient scenarios: an evolving tool in psychiatric education. Academic Psychiatry. 2012; 36(2):146. | Yes |  |
| Silva RD, Albuquerque SG, Muniz AD, Filho PP, Ribeiro S, Pinheiro PR, et al. Reducing the schizophrenia stigma: a new approach based on augmented reality. Computational intelligence and neuroscience. 2017; 2017(1):2721846. | Yes |  |
| Sperling JD, Clark S, Kang Y. Teaching medical students a clinical approach to altered mental status: simulation enhances traditional curriculum. Medical education online. 2013;18:1–8. | No | Not specific to mental health |
| Una LB, Brangman S, Indelicato A, Krueger A, Ludwig A, Slutzky AR, et al. Using second life to teach health professions students about Alzheimer’s Disease: A comprehensive review. Gerontology & geriatrics education. 2023;44(2):243–53. | No  Note: from reference screening/citation searches | Not primary research |
| Vallance AK, Hemani A, Fernandez V, Livingstone D, McCusker K, ToroTroconis M. Using virtual worlds for role play simulation in child and adolescent psychiatry: An evaluation study. BJPsych Bulletin. 2014;38(5):204–10. | Yes |  |
| Woon LS, Mohd Daud TI, Tong SF. “It kinda helped us to be there”: students’ perspectives on the use of virtual patient software in psychiatry posting. BMC Medical Education. 2023;23(1):851. | Yes |  |
| Yu JH, Chang HJ, Kim SS, Park JE, Chung WY, Lee SK, et al. Effects of high-fidelity simulation education on medical students’ anxiety and confidence. PLoS ONE [Electronic Resource]. 2021;16(5):e0251078. | No | No educational outcomes |
| Zackoff MW, Young D, Sahay RD, Fei L, Real FJ, Guiot A, et al. Establishing Objective Measures of Clinical Competence in Undergraduate Medical Education Through Immersive Virtual Reality. Academic Pediatrics. 2021;21(3):575–9. | No | Not specific to mental health |
| Zare-Bidaki M, Ehteshampour A, Reisaliakbarighomi M, Mazinani R, Ardakani MRK, Mirabzadeh A, et al. Evaluating the Effects of Experiencing Virtual Reality Simulation of Psychosis on Mental Illness Stigma, Empathy, and Knowledge in Medical Students. Frontiers in psychiatry Frontiers Research Foundation. 2022;13:880331. | Yes |  |
| Zlotos L, Power A, Hill D, Chapman P. A Scenario-Based Virtual Patient Program to Support Substance Misuse Education. American Journal of Pharmaceutical Education. 2016;80(3):48. | No | Not undergraduate medical student participants |
