# Supplementary material for: Virtual and Augmented Reality in Undergraduate Medical Education in Psychiatry: A Systematic Review
Source: Clin Teach. 2025 Jun 18;22(4):e70128. doi: 10.1111/tct.70128 (PMC12175210; doi:10.1111/tct.70128)
Supplement: Supplementary file 4 — Table S2. Summary of study characteristics and results. [file TCT-22-e70128-s001.docx]

| **Supplementary table 2: summary of study characteristics and results** | | | | | | | | | | |
| --- | --- | --- | --- | --- | --- | --- | --- | --- | --- | --- |
| **Author, year and country** | **Objective** | **Participant description** | **Design** | **Comparator** | **VR/AR setup** | **Subject taught** | **Other learning strategies** | **Outcome measures** | **Results** | [**MERSQI**](https://pubmed.ncbi.nlm.nih.gov/26107881/)**/ROB (RCT)** |
| Bard et al., 2023 (UK) | To determine feasibility and efficacy of VR as a learning tool to generate understanding and empathy for people living with dementia and their families | n=150 year 2 medical students | 45 minute intervention, pre and post survey using modified likert scale tp assess self-reported emotions and attitudes. 3-module lab takes students through progression of a woman diagnosed with AD through mild, moderate and severe stages. Included alterations in auditory processing. | No control group | Immersive first-person VR experience. | Understanding of lived experience of dementia | No associated learning activity specified | Level 1 Reaction (emotions, self-reported attitudes towards VR as learning tool) Level 2 Attitudes (5-point Lickert scale .on self-reported level of understanding). | Significant difference in pre- and post-test scores on all 4 questions on understanding of experience of living with dementia as patient and caregiver. 113/149 said they would like to have more VR experiences incorporated into the curriculum. After the VR, 86.7%, 94% and 91.3% said that the VR experience affected them emotionally, had made them consider how they would approach patients, had given them a better understanding of impact of dementia on people and families. | MERSQI 8.5  ROB n/a |
| Dupuy et al., 2019 (France) | Use of VP interview, simulating major depression, to assess diagnostic skills and empathy in medical students and their attitude to VP | n=35 4th year medical students at Bordeaux university | Students interacted with a VP simulating depression whilst being monitored for verbal and non verbal reactions. They answered MCQ style questions relating to depressive psychopathology and were scored on empathy based on question choice and objective emotion recognition software. Post test qualitative element with student attitude towards VP | No control group | VP simulation of major depression where students interacted via microphone simluating a real clinical interview and MCQ questions, around 35 minutes | Diagnosis of depressive disorders and student empathy towards depression | No associated learning activity | Level 2: Knowledge and attitudes (Self-reported knowledge and attitudes and beliefs in semi structured interview) Level 1: empathy reaction from standardised software measuring emotional reactions | Overall empathy and diagostic scores were high. Scores were significantly lower for semiology MCQs (t(68) = 3.489; p < .001) and students made  significantly more errors during semiology MCQs than in empathy questions (t(68) = 8.064; p <.001). Qualitive data showed positive responses for use of VP as educational tool, ease of use and realism. Some found the questions too easy. | MERSQI 11  ROB n/a |
| Fitzmaurice et al., 2007 (Ireland) | To evaluates attitudes towards using a web-based interactive video interview simulator | n=189 medical students during psychiatry placements in years 3,4 or 5 | Pre and post survey of attitudes towards using a web-based interactive video interview simulator | No control group | Web-based interactive video interview simulator, automated with no facilitator required, interactive scenarios | Psychiatric interview skills in depression | No associated learning activity | Level 1 Reaction | Response to question that web resources useful was 20% at baseline and 76% at f/u. 55% felt that they were "virtually interviewing a patient". Focus group discussion - students agreed that further modules simulating different disorders would be of benefit to their skills development | MERSQI 5  ROB n/a |
| Fleming et al., 2009 (UK) | To test the ability of an off-the shelf virtual reality simulation system to improve the clinical skills of students and primary care physicians | Medical students and other healthcare professionals and students at University of Wisconin-Madison. n=102 total, n=36 medical students | Participants recruited by email invitation. 3 standardised cases (trained SP) each for pre and post test. Simulations related to alcohol screening and intervention. Participants in control group expected to read materials and practice simulation ≥10 times during 3-month study period. Follow-up was at 6 months. | No learning activity | VR. Used SIMmersion simulation technology (SIMmersion LLC, Columbia, MD). Automated with no faciliator required. Interactive scenarios. Learners chose questions to ask VP from prespecified selection, programmed response from VP. Feedback provided to learner about appropriateness of questions asked during and after simulation. | Alcohol screening and brief intervention. | No associated learning activity | Level 2 Skills | Significant increase in skills scores for alcohol screening (p<0.001) and brief intervention skills (p<0.04) for intervention group compared to control. | MESQI 13.5  ROB High |
| Foster et al., 2014 (USA) | To assess whether medical students respond empathetically with VP and does this vary with level of training. To assess efficacy of VP as undergraduate teaching tool | n=155 medical students across 3 years of undergraduate study in a 2 year period | Interaction with 2 VP txt based web design. Transcripts of student interactions with VP were analysed by applying the ECCS (empathic communication coding system). First part codes empathic opportunities presented VP (3 categories: emotion, progress, challenge). Second part codes responses by 1 - 7 hierarchical levels | No control group | medical students interacted with 2 VP (depression and bipolar disorder) created in 'Virtual People Factory', webbrowser interface where students interact via text responses with VP which is a static image | empathy towards VP with depression and bipolar | No associated learning activity | level 2: skills (7 point hierarchy scoring system for empathy on ECCS) | Mean empathic intensity of responses was 1.5 (0-6 scale) for depression and 2.2 for bipolar. 3rd year students responded to depression VP with significantly more empathy than 1st (p<0.001) and 2nd year students (p<0.001). students communicated empathically with VPs, but showed less empathy than physicians interacting with live patients | MERSQI 13  ROB n/a |
| Foster et al., 2015 (USA) | To determine whether students interacting with bipolar VP who attempts suicide were as likely as those completing a video teaching module to assess suicide risk | n=67 year 2 medical students at the Medical College of Georgia, n=67 | RCT. One arm was interaction with virtual patient using web-based browser system | Watching video of clinician interaction with patient with bipolar disorder who models suicide risk assessment, with formal case history presentation | Web-based virtual patient system (Virtual People Factory, VPF), automated with no facilitator required. Interaction by typing, static image of patient on screen. Database of responses that are selected by system as appropriate response to student question | Suicide risk assessment in bipolar disorder | No additional learning activity | Level 2: skills: Assessed interview with SP who rated and was blinded to VP/video allocation. 19-item suicide assessment checkliston suicide risk and bipolar symptomatology, 14-item communication checklist, 5 items from rapport subscale of Medical Student Interviewing Performance Questionnaire. Level 1: reaction - student satisfaction | VP group asked 4 of 5 suicide risk questions more often and 11 of 14 bipolar symptom areas, differences not statistically significant, small effect sizes in favour of SP. Students preferred video over VP as educational tool (p=0.007). 17/18 of VP students said that simulation of real life was good or excellent (11 average, 8 fair, 5 poor). | MERSQI 14.5  ROB High |
| Foster et al., 2016 (USA) | To determine whether virtual patient interventions enhance empathy in subsequent student encounters with human patients | n=70 year 1 medical students at Medical College of Georgia, n=70 | RCT. Interview with 21 year old VP with depression. Empathy feedback from human assessor available online after session. | 2 control groups, one with noninteractive video vignettes illustrating a backstory and another with a control VP which was a typed interaction with the depressed VP without empathy feedback or patient backstory. | Interviews with virtual patient with online text-based interface. Natural language processing algorithm used to allow VP response to questions. | Empathy in interaction with patient with depression | No additional learning activity | Level 1: Reaction (5 point scale of satisfaction with VP interaction - poor (1), fair, average, good, excellent (5)) Level 2: Skills - based on SP interview - Empathic Communication Coding System Assessor - assessors were trained and blinded. Standardized patient communication and symptom (history taking skills) checklists by SPs, Medical Student Interview Performance Questionnaire (MSIPQ) | Significant increase in overall empathy scores in intervention group compared to backstory group but not control group. Significant increase for 2 communication skills items for intervention and backstory groups compared to the control VP group. Participant report of overall satisfaction with the VP interaction was reported as mean 3.3 (1.0) and median 3.0 (2.0). | MERSQI 13.5  ROB High |
| Gilmartin-Thomas et al., 2018 (Australia) | To evaluate the impact of a virtual dementia experience on self-reported attitudes and knowledge towards people with dementia | n= 41 year 3 medical students and n= 42 year 4 undergraduate pharmacy students | Focus groups with students after taking part in intervention arm of a non-randomised controlled study | None | 1.5 session where partipants engaged in a group activity that required communication and recall whilst exposed to multisensory virtual simulation of communication and memory difficulties associated with dementia. Pbservation of an actor demonstrating effective and ineffective communication strategies. | Experience of living with dementia | Reflection and discussion to consider approaches to dementia care and support and the challenges faced by people living with dementia | Level 2: Knowledge and attitudes (Self-reported knowledge and attitudes and beliefs in focus group) | 29 medical students and 24 pharmacy students took part in focus groups. Students reported that the experience was impactful and provided insight into the experience of some symptoms of dementia.They also identified ways in which their future practice could be more dementia-friendly. | MERSQI 6.5  ROB n/a |
| Gilmartin-Thomas et al., 2020 (Australia) | Comparison of virtual dementia experience to vs standard curriculum on knowledge and attitudes towards people with dementia | n=69 year 3 medical and 229 year 4 pharmacy undergraduate students. n=80 intervention, n=198 control | Non-randomised controlled trial. Medical students allocated to groups based on tutor groups, pharmacy students who volunteered became intervention group, non-volunteers were control. | Standard curricular teaching | 1.5 session where partipants engaged in a group activity that required communication and recall whilst exposed to multisensory virtual simulation of communication and memory difficulties associated with dementia. Observation of an actor demonstrating effective and ineffective communication strategies. Followed by facilitator-guided personal reflection and follow-up group discussion. | Experience of living with dementia | Facilitator-guided personal reflection and follow-up group discussion. | Level 2 Attitudes: 20-item Dementia Attitudes Scale - Likert-type scale responses (1 "strongly disagree" -7 "strongly agree") completed at baseline and after intervention to assess cognitive and affective components of attitude. | Paired baseline and control questionnaires available for n=64 medical students and n=214 pharmacy students. Greater change in DAS scores in intervention group (p<0.05) compared to control for combined medical and pharmacy student group. For medical students only, greater change in DAS score in intervention group for 16/20 DAS items, with significant improvement in total score and subdomains of comfort and knowledge. | MERSQI 10  ROB n/a |
| Hayes-Roth et al., 2004 (USA) | To assess the effectiveness (change in skills and attitude) and efficiency of using virtual coach and virtual patient workshops to teach brief intervention skills In alcohol misuse | n=31 medical and nursing students, medical students from Stanford university | Semi-randomised control trial. Three groups including intervention (STAR workshop with virtual coach and virtual patients teaching brief intervention skills through target protocol based on motivational interviewing), e book and control group (no training). Pre and post interventions tests of knowledge and skills and 2 week follow up with real simulated patients during phone intervention to assess skills | E-Book group and No learning activity group (Control) | Workshop with online virtual coach and interactions with 3 virtual standardised patients | Brief intervention skills for alcohol misuse based on motivational interviewing techniques | No additional learning activity | Self-report of attitude on 5-point Likert scale, skills assessed on post-test short answer probes and objective 2 week delayed telephone intervention with live standardised patient which was scored against target protocol | 100% of STAR subjects improved over pre-training skills immediately after intervention and averaged 89% correct responses post assessment. Retained skills test 2 week post intervention showed STAR subjects performed substantially better than E-Book and Control subjects. 100% of STAR subjects improved over pre-training skills. STAR subjects averaged 89% correct responses on delayed skills probes, with 46% scoring >90%. | MERSQI 11.5  ROB n/a |
| Jachna et al., 1993 (USA) | To assess the effectiveness (pre- and post-simulation MCQ scores) of a using a computerised case simulation to teach consultation psychiatry to medical students | n=38 3^rd^ year medical students at Yale-New Haven Hospital | Pre- and post-simulation test consisting of 10 multiple-choice, single answer questions (5 pertaining to general psychiatry and 5 pertaining to specific learning points covered in the simulation). Students completed the computerised case simulation in groups. | None | Computerised case simulation using static images selection of preset on-screen statements or responses | Identification, diagnosis and management of alcoholism, depression and PTSD | No additional learning activity | Level 2: knowledge: pre- and post-intervention test of knowledge pertaining to general issues in concultation psychiatry as well as specific learning points covered in the intervention | Students scored significantly better on both questions related to general issues in consultation psychiatry and questions related to specific learning points covered in the simulation on the post-simulation test. 67% of answers to questions on learning points covered in the simulation were correct on the pre-simulation test compared to 81% on the post-simulation test (p=0.005). 78% of answers to questions on general psychiatry were correct on the pre-simulation test compared to 86% on the post-simulation test (p=0.025). | MERSQI 13  ROB n/a |
| Matsumura et al., 2018 (Japan) | Comparison of virtual dementia experience to vs standard curriculum on knowledge and attitudes towards people with dementia | 5th year medical students at Kagawa University. n=79 (36 intervention, 43 control) | Non-randomised controlled trial. One year group of students compared to previous year group in terms of acquisition of knowledge about dementia. Pre- and post-intevention tests of knowledge of dementia were completed in addition to a questionnaire based on the Attention, relevant, confidence and satisfaction motivational model for teaching development. Students in intervention group had similar lectures to control group but less patient contact than control group. | Students from the cohort who completed the same year of study the previous year. | Interaction with 4 different VPs with different presentations of dementia in simulated 3D examination room on a computer screen. The virtual platform included an electronic record system and feedback regarding students' choices and actions. Student choices were indicated by pressing relevant buttons. | Dementia | No additional learning activity | Level 2 (knowledge) assessed using pre- and post-intevention tests of knowledge of dementia using tool developed by authors with content validity assessed by psychiatrists and internal consistency 0.72 using Chronenbach's alpha. Level 1 reaction: Attention, relevant, confidence and satisfaction motivational model for teaching development. | No difference between control and intervention group knowledge scores at baseline. Post-test scores for intervention group higher than control group (p=0.01). Motivation scores in experimental group increased post-intervention in all 4 categories. | MERSQI 13.5  ROB n/a |
| Pataki et al., 2012 (USA) | Assessing feasibility of using a virtual patient in child and adolescent mental health training using VP with PTSD | n=7 medical students, n=4 psychiatry residents, n=4 child psychiatry residents at the University of Southern California | Pre-and post intervention test of knowledge of PTSD and questionnaires related to VR and VP | No control group | 15-20 minute clinical interview in form of verbal dialogue with virtual adolescent patient with PTSD visible on computer screen. | PTSD assessment | No additional learning activity | Level 2: knowledge: Pre-and post intervention test of knowledge of PTSD. Level 1 reaction: Pre-assessment scales of openness to experiences, immersive tendencies, experience of VR. Post-assessment Presence Questionnaire (mastery/response/control related to environment experienced) and VP believability questionnaire. | Limited data presented. No change in knowledge pre and post-test. Students indicated positive feelings about VP experience although sometime found her frustrating to interview. | MERSQI 10  ROB n/a |
| Rampling et al., 2012 (UK) | Assessing students perception of virtual patient as an educational tool and effect on stimulating interest in psychiatry | n=24 students from 3 cohorts of 50 medical students at St Georges University London | Initial test with 3 students then 150 students emailed with instructions. 20 students completed as part of PBL tutorials with facilitator.Post test survey on attitudes towards educational value of virtual patient and later free text responses. Post test survey for knoweledge and clinical usefullness | No control group | Interaction with VP in 'Second life' online virtual world interviewing patient with psychosis inlcuding interacting with the environment, communicating via chat interface with text responses and triggered audio responses to some questions | Psychosis knowledge and clinical usefulness | No additional learning activity |  | Free responses showed disadvantages outweighed advantages for using as educational tool including being too slow, hard to navigate and not realistic. Post test survey did not show improvement in knowledge or clinical usefullness such as improving confidence or history taking skills | MERSQI 6  ROB n/a |
| Shah et al., 2012 (USA) |  | n=67 3rd year medical students at Georgia university Second cohort included 1st and 2nd years medical students | Initially 67 3rd year students attended lecture of psychiatric interviewing followed by online interaction with VP, then completed a questionnaire rating the tool on a 5-point Likert scale. A follow-up cohort of 1st and 2nd year medical students then underwent the same protocol using a more sophisticated version of the VP. | No control group | Online platform 'Virtual People Factory' involving VPs with instant messaging function | Major depression | No additional learning activity | Self-reported attitude towards VP on 5-point Likert scale. Elicitation of discoveries (patient signs/symptoms) compared between year groups. |  | MERSQI 10.5  ROB n/a |
| Silva et al., 2017 (Brazil) | To determine the impact of an augmented reality intervention on attutudes towards a fictitious patient with schizophrenia | n=21 medical students from 3 universities in the state of Ceara who responded to invitation to participate | Pre-and post intervention questionnaire on stigma, post-intevention quesitonnaires on the experience of the virtual environment | No control group | Augmented reality - use of Sony HMZ-T2 glasses to simulate visual and auditory hallucinations | Experience of positive symptoms in schizophrenia | No additional learning activity | Level 2 Attitudes: Pre and post-intervention stigma questionnaire developed by authors. Level 1 reaction: Post-intervention questionnaire about experience of the virtual environment Ilickert-style scale 1 (little)-5 (extremely)) and any adverse effects experienced (yes/no responses to list). | Trend post-intervention to want to help fictitious patient, also to hospitalise and treat involuntarily. Increase in empathy but also seeing the presentation as more serious and the patient as more dangerous. Increase in mean stigma score (decreased stigma) from 32.05 to 35.38 (p=0.004) with statistical significance in pity, fear and segregation. 38.1% reported blurry vision and eyestrain, 19% reported general malady, "heavy head" and weariness, all side effects minor and short-lived. Positive responses from >80 % of students on most items related to experience of the simulation. | MERSQI 8.5  ROB n/a |
| Vallance et al., 2014 (UK) | To explore attitudes towards a teaching session in psychiatry delivered in a virtual world (Second Life) | n=10 year 4 4th year medical students at Imperial College London who responded to internal advertisement | Single arm with post-intervention qualitative and quantatitative evaluation of teaching tool | No control group | 90 minute teaching session in Second Life in virtual clinic with users interacting through avatars. Session included briefing, role play, reflective and debriefing stages | Psychiatric assessment and management, communication and professional skills. | No additional learning activity | Level 1 Reaction: Qualitative and quantitative evaluation of the learning technology. Questionnaire developed by the authors and adapted, included adapted version of a related questionnaire on "computer gaming". Items grouped into "affective", "behaviour", "perceived control" and "perceived usefulness". | Males and people with more experience with onliine multiplayer games scored higher on being "able to make the computer do what I need it to do while learning using second life". Higher scores related to simulation fidelity were associated with higher scores across all 4 domains and on "general attitude". Thematic analysis (from 7 participants in focus group): participants valued skills development, and identified advantages of standardising clinical experiences, expanding clinical exposure, mimic illness (e.g. clothing in mania, thinness in anorexia nervosa), distance learning, saving money on communiting. Limitations identified in thematic analysis were recognition that "nothing replaces the real thing" and limited "added value" compared to real-life role play or other learning opportunities. | MERSQI 9  ROB n/a |
| Woon et al., 2023 (Malaysia) | A qualitative study to explore medical students' learning experiences with a virtual patient through thematic analysis | 10 4th year medical students at the National University of Malaysia that had completed their psychiatry posting using DxR Clinician | Students completed a modified TBL module using a VP on the DxR Clinician platform in two groups of five. Students then participated in an online focus group to give feedback on their learning experience after using DxR Clinician | None | Online TBL module completed in groups using a VP designed using DxR Clinician. | Dementia history taking | No additional learning activity | Self-reported learner experience of using VR | Thematic analysis identified three main themes: fulfilling desired pedagogy, realism of the clinical case and ease of use related to technical settings. In general, students appreciated getting instant feedback during the module but found the group work to be time-consuming. Some students felt aspects of the VR platform simulated real life but found that the pre-listed questions and text responses were not a good substitute for real patient interaction. In general, students found the platform difficult to navigate and use. | MERSQI 7  ROB n/a |
| Zare-Bidaki et al., 2022 (Iran) | Comparison of effect of virtual reality simulation of psychosis (VRSP) to routine education (visiting patients under supervision) on stigma, empathy and knowledge in undergraduate medical students | n=144 2nd and 3rd year undergraduate medical students and the Birjant University of Medical Sciences | Cluster randomised controlled trial of single session of VRSP vs control, with pre and post intevention questionnaires and telephone follow-up at 1 week and 1 month | Visiting patients on the ward under supervision as routine practice | VR headset with scenario that mimics auditory hallucinations and delusions of reference. | Positive symptoms of schizophrenia | Lecture about positive psychotic symptoms | Pre-intervention, after lecture and after intervention: Objective assessment of knowledge (designed by authors) questionnaires on stigma (Persian version of "Mental illness stigma by world psychiatric association", empathy (Persian version of the Jefferson Scale of Physician Empathy), and side effects. Side effects also assessed at 1 week and 1 month post-intervention). Objective assessment of knowledge pre and post-intervention | VSRP associated with significant increase in scores on knowledge and empathy and reduced stigma. Short-lived minor side effects reported by small number of participants. | MERSQI 13.5  ROB High |
